# Micro/Nano hierarchical peony-like Al doped ZnO superhydrophobic film: The guiding effect of (100) preferred seed layer

**DOI:** 10.1038/srep19187

**Published:** 2016-01-12

**Authors:** Yang Li, Jingfeng Wang, Yi Kong, Jia Zhou, Jinzhu Wu, Gang Wang, Hai Bi, Xiaohong Wu, Wei Qin, Qingkun Li

**Affiliations:** 1 Department of Chemistry, Harbin Institute of Technology, Harbin, Heilongjiang 150001, PR China; 2 School of Materials Science and Engineering, Harbin Institute of Technology, Harbin, Heilongjiang 150001, PR China; 3 Key Laboratory of Electrical Engineering, College of Heilongjiang Province Major Laboratories of Integrated Circuits, Heilongjiang University

## Abstract

In this communication, we present a versatile and controllable strategy for formation of superhydrophobic micro/nano hierarchical Al doped ZnO (AZO) films with a water contact angle (CA) of 170 ± 4°. This strategy involves a two-step layer-by-layer process employing an atomic layer deposition (ALD) technique followed by a hydrothermal method, and the resulting novel AZO surface layer consists of (100) dominant nano-rice-like AZO seed layer (the water CA of 110 ± 4°) covered with micro-peony-like AZO top. The growth mechanisms and superhydrophobic properties of the hierarchical AZO layer are discussed. It is believed that the present route holds promise for future success in the design and development of practical superhydrophobic materials.

Superhydrophobic materials and coatings with water contact angle larger than 150° have been extensively investigated due to their wide applications from housewares to industrial products^1^,^2^. For instance, solar cell panels, goggles and windows for photoelectronic devices generally demand their surfaces with properties of self-cleaning, anti-fog and superhydrophobicity^3^. The key points of superhydrophobic states include the surface energy of the chemical composition and the geometrical rough structure of solid surfaces^4^. Owing to the lower surface energy, the organic perfluorinated materials turn into the most common hydrophobic material, however those organic coatings are costly and unstable for the application. Therefore, increasing attentions are drawn on some inorganic materials with specific hierarchical rough surfaces^5^,^6^.

Al-doped ZnO (AZO) materials have attracted considerable attentions owing to their unique optical and electrical properties, such as wide band gap and high exciton binding energy^7^,^8^,^9^. AZO film can provide valence ions and higher carrier mobility, making it a desirable candidate for various photovoltaic and solar cell applications^10^,^11^. Therefore, the superhydrophobic AZO intelligent materials are potential and promising in the photoelectric fields.

To fabricate the required superhydrophobic surface roughness of AZO film, various methods have been proposed in literatures. A two-step layer-by-layer approach were adopted in several research groups^12^,^13^. AZO seed layer was deposited on a substance by solution method, sol–gel method, magnetron sputtering methods and others, which can lower the activation barrier for the following nucleation and enhance the film adhesive force with top layer. Micro-nano AZO structures were fabricated subsequently using the hydrothermal method, plasma etching, and vapor deposition on the seed layer^14^,^15^. It is worth fabricating the special and micro/nano hierarchical AZO structure surface, by enhancing the surface roughness, so that superhydrophobic AZO films materials can be achieved, without involving the perfluorinated material.

In most research works, the surface of ZnO micro-nanorods arrays grown based on (002) preferred seed layer is still hydrophilic, and thus the fluorination method needs to be used for formation of the superhydrophobic surface texture^16^. In contrast, other orientation dominant seed layer tends to induce more complex micro nanostructure for superhydrophobic surface^17^. In this case, the seed layer obviously plays a subtle guiding role in controlling the micro-structure for the subsequent growth process. Therefore, the growth method, orientation and guiding effect of seed layer should be investigated to further discuss the morphology and the superhydrophobic feature of the generated AZO film.

Herein, we present a facile strategy to fabricate superhydrophobic AZO film using an atomic layer deposition (ALD) technique and hydrothermal methods. In our well-designed process, at first, nano rice-structured (100) preferred AZO thin layer with a thickness of ~20 nm *in-situ* grows on a glass substrate using the ALD technique. Subsequently, the micro-flower-structure AZO layer continuously grows on the seed layer using the hydrothermal method. The resulting micro-nano hierarchical structure of AZO film is superhydrophobic and nearly transparent without any further treatment. Our approach is much simpler and more feasible than the reported ones using the expensive fluorochemicals to generate superhydrophobic surfaces. Owing to the flexibility of the ALD technique, the facile two-step strategy introduced here is a promising candidate in the photoelectrical applications such as flexible photovoltaics and displays.

## Results and Discussion

**Figure 1:**
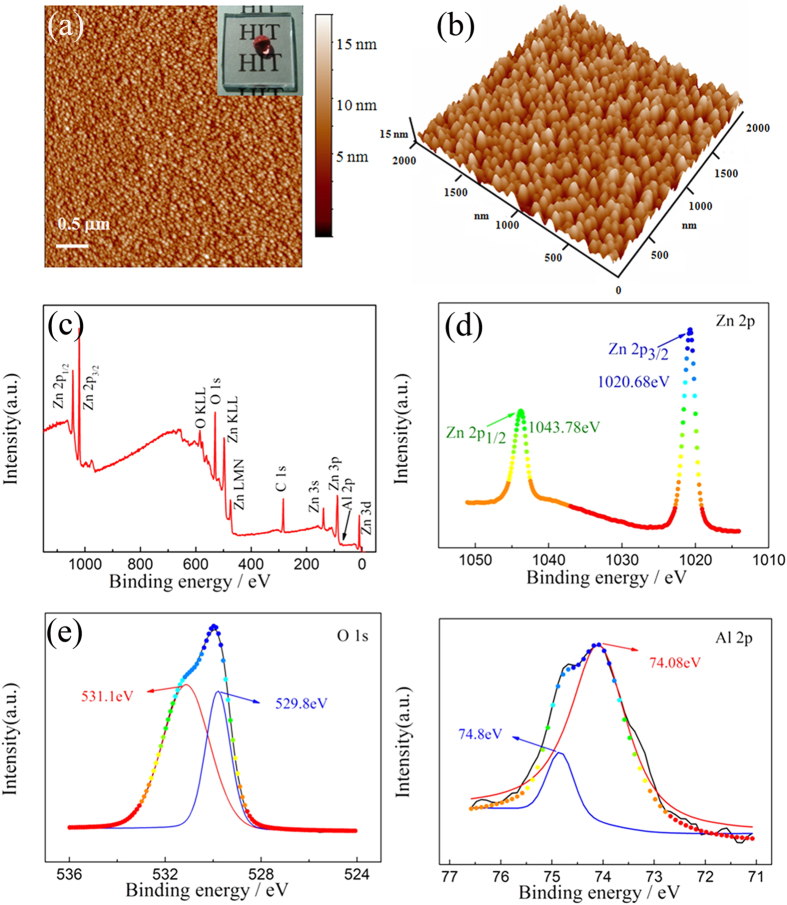
AFM images of AZO seed layer thin film, scanning area: 2 μm × 2 μm, 2D image (**a**) and 3D image (**b**). Inset in (**a**) is digital image of AZO seed layer on glass substrate. (**c**) Survey XPS spectrum of the AZO seed layer. (**d–f**) XPS spectrums of the Zn 2P, O1s and Al 2P core level regions and their Gaussian-resolved results, respectively.

The strategy for preparing the hierarchically structured superhydrophobic AZO thin films is schematically presented in Figure S1. The AZO seed layer was formed on the silica and glass substrates through deposition using the ALD method. Figure 1a,b show the 2D and 3D AFM images of AZO seed layer film with the thickness of 20 nm on the silica substrate. The seed layer consists of the arrays of uniform nano-rice-like protuberances with acicular points, showing the gain size and roughness (rms) values of 10 nm and 3 nm, respectively. The seed layer should be thick enough to allow subsequent growth of homogenous AZO crystal by the hydrothermal method. In this study, we controlled the thickness of the seed layer around 20 nm, which presents good stability for the subsequent treatment and high transparency. The novel AZO seed layer with the nano-rice array morphology on the glass substrate shows hydrophobic against a spherical water droplet with a water CA of 110 ± 4°. Similar features have been found in nature, such as lotus leaves and the cicada’s wings^18^. The transparency of the seed layer is very close to the bare glass substrate (Insert Fig. 1a). The transmittance of the AZO 20 nm seed layer is about 95% (Figure S2).

The survey XPS spectrum of the AZO seed layer is shown in Fig. 1c, which shows the representative peaks of Zn and O. Figure 1d–f demonstrate the high resolution XPS spectra of the Zn 2p, O1s, and Al 2p core levels and their Gaussian-resolved results for the AZO seed, respectively. The Gaussian-resolved result for Zn 2p spectra in Fig. 1d shows two main peaks centered at 1020.68 eV and 1043.78 eV, which were assigned to Zn 2p_3/2_ and Zn 2p_1/2_ states respectively^19^. The Gaussian-resolved result for O1s spectra in Fig. 1e shows two components of oxygen varying in chemical states which are different in binding energy. The lower binding energy component, centered at 529.8 eV, is attributed to O^2−^ ions on the wurtzite structure of hexagonal Zn^2+^ ion array^20^. Meanwhile, the high binding energy component located at 531.1 eV is associated with O^2−^ in the oxygen deficient regions within the matrix of ZnO^21^. Figure 1f indicates the presence of Al^3+^ in the seed layer according to the Al 2p peak, which can be Gaussian-resolved with two peaks centered at 74.08 eV and 74.8 eV. The 74.08 eV obviously suggested that the Al^3+^ had substitutionally incorporated into Zn^2+^ sites. In addition, the higher energy 74.8 eV is associated with the stoichiometric Al_2_O_3_, indication of generation of Al_2_O_3_ within the ALD growth process^22^. It can be found that the atomic concentration of Al is 3.43% for the AZO seed layer. In addition, the distribution of Al dopants in the surface and the whole sample were also investigated using energy-dispersive x-ray spectroscopy (EDS) in Figure S2 and inductively coupled plasma atomic emission spectroscopy (ICP-AES), respectively. The Al atomic dopants of 3.41% and 3.01% had been obtained for the surface layer and longitudinal direction in the ALD AZO film. The Al dopant concentration for the whole sample in ICP-AES was slightly lower than that in the surface in the XPS and EDS analysis. This behavior can be attributed to the ALD AZO film growth process, in which each cycle ended up with 19 Zn: 1 Al: O sequence. Therefore, the Al dopant was more abundant in the surface layer than that in the whole sample, and the similar results have also been obtained by other groups^23^.

The seed layer with specific nanostructures, excellent transparency and ion doping modification were successfully obtained in the first step. Then, upper AZO micro-flower thin layers were fabricated via the short time hydrothermal method.

**Figure 2:**
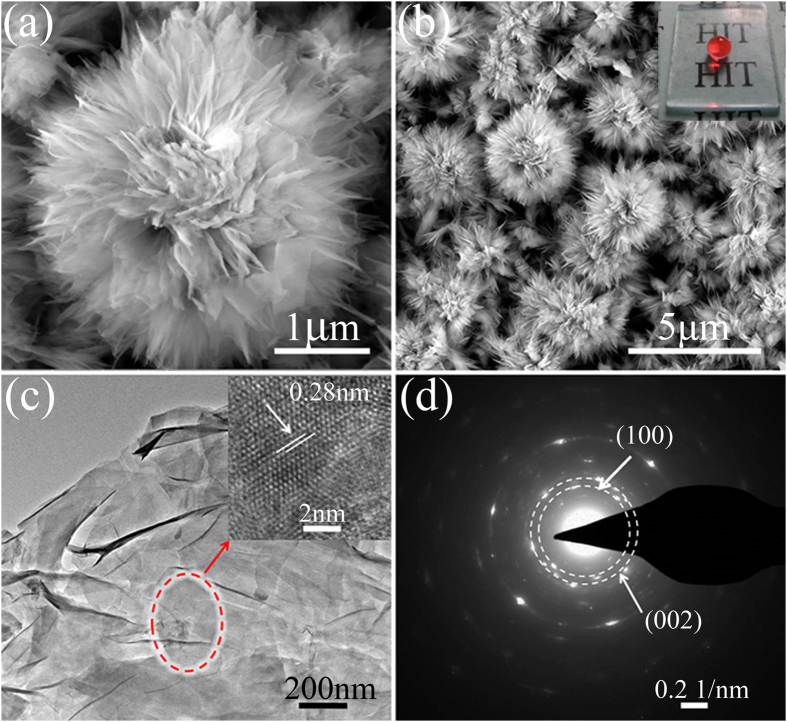
FE-SEM top-images of the as-prepared AZO films at high magnification (**a**) and low magnification (**b**). Inset in (**b**) is digital image of AZO films on glass substrate. (**c**) TEM image and high-resolution TEM image (insert of Fig. 2c,d) selected area electron diffraction (SAED) of AZO petal.

Figure 2a,b are SEM top-images of the hierarchical AZO hydrothermal film at low and high magnifications. It can be seen that the diameters of AZO micro-flowers are about 2 ~ 3 μm, showing a uniform peony-like structure with dense and thin nano-petals. The unique micro/nano flower-like AZO layer presents excellent superhydrophobicity and good transparency, as indicated by the clear letters underneath the glass slide coated with this layer (Insert Fig. 2b). Figure S3 illustrates the transmittance spectra of the AZO seed layer and the hydrothermal layer. The average transmittance values of the AZO hydrothermal layer are about 80%, which is suitable for the practical optical device. The optical band gap (E_*g*_) of the AZO seed layer and the hydrothermal layer were estimated by Tauc relation 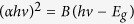. Where α, *hv* and E_*g*_ is the optical absorption coefficient, the photon energy and the energy gap, respectively. Based on the transmittance spectra, the (*αhν*)^2^ versus *hν* graph was plotted in Figure S4, in which a straight line is fitted for the straight region. The extrapolation of this straight line to *x* axis gives the value of the band gap for AZO films. The results indicate that AZO seed and hydrothermal layers both exhibit higher E_*g*_ values ~3.7 eV than undoped ZnO (3.36 eV), which is attributed to Moss-Burstein effect, caused by an increase in free electron concentration due to Al doping^24^.

The micrographs of the hierarchical AZO film were taken by TEM and high-resolution TEM (HRTEM). Figure 2c depicts bright field TEM and HRTEM images of certain nano-petals of the AZO flower. From the HRTEM observation, it can be found that there are AZO polycrystalline grains with the distances of crystalline planes of 0.28 nm (Insert Fig. 2c), which are corresponding to the interplanar spacing of (100) planes of AZO. The selected area electron diffraction (SAED) image is shown in Fig. 2d. The presence of diffused rings and regular spots further confirms the above results.

**Figure 3:**
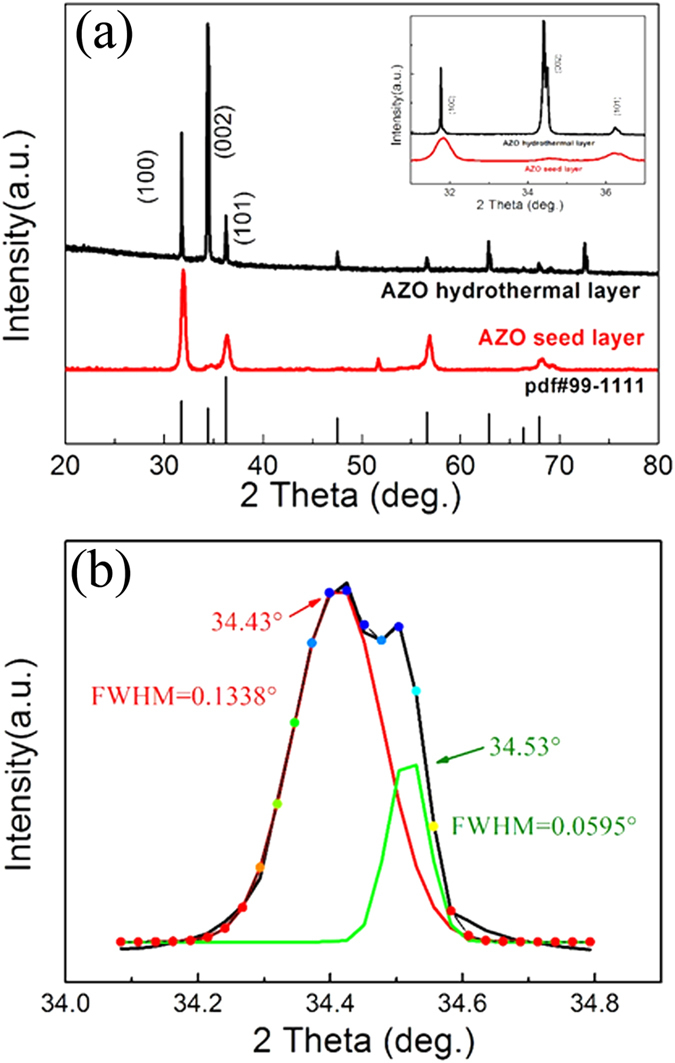
(**a**) X-ray diffraction of the AZO seed layers and the AZO hydrothermal films; Inset shows the zoom-in view of 31–37 deg. (**b**) The magnification and Gaussian fitting results of (002) diffractive peak of the AZO hydrothermal films.

Besides the transparency and surface morphologies, the crystallographic structure and dominant orientation of the seed layer are significant and critical for guiding growth of AZO top layer during the second step. Owing to the island-like growth mechanism of AZO during the ALD process, heat treatment was not adopted^25^. The crystallographic structures of the as-prepared AZO seed layer (lower) and the AZO hydrothermal film (upper) were characterized by using XRD, as shown in Fig. 3a. The results indicate that the AZO seed layer possesses a wurtzite crystal structure according to the standard data (JCPDS, No. 99–1111) with (100) preferred plane, different from (002) dominant ZnO thin layer prepared using other methods like the sol-gel and magnetron sputtering methods^26^. There are no extra peaks related to aluminum metal and those from other zinc aluminum phase, indicating that the Al ions substituted the Zn sites without changing the wurtzite structure. In the second hydrothermal step, the AZO top layer sequentially grows and evolves restrictedly guided by the (100) crystallographic planes of the former seed layer. Because of the different hierarchical structures and surfaces tensions of the two layers, the diffraction peaks of the AZO hydrothermal film were slightly shifted compared with those of the AZO seed layer (Insert Fig. 3a).

It can be seen that the (002) peak of the AZO hydrothermal film can be decomposed to two peaks at 34.43° and 34.53° by Gaussian fitting. As shown in Fig. 3b, 34.43° is the typical (002) diffractive peak of ZnO wurtzite crystal. The (002) peak splitting at 34.53° can be inferred the lattice disorder of the AZO crystal. The morphology of the peony-like AZO structure with dense and thin nano-petals (Fig. 2a) is a possible reason for the disorder of crystal structure^27^.

The chemical states of the compositional elements in AZO hydrothermal film were investigated by the XPS and the survey and representative Al 2p spectra are shown in Figure S5. The XPS results reveal that the alumina concentration was equal to 3.08% after hydrothermal process.

**Figure 4:**
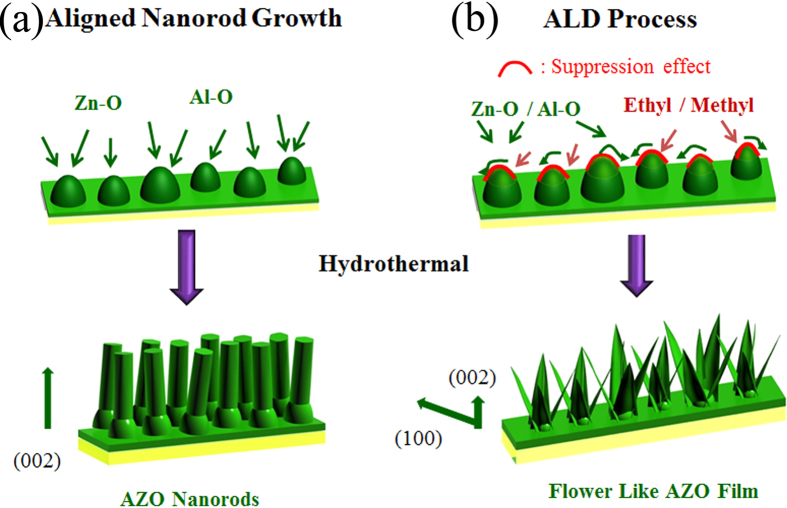
Schematic of plausible mechanism of (100) dominant planes in the seed layer and the formation of AZO architectures for (**a**) AZO nanorods arrays and (**b**) AZO flower-like structure.

The formation mechanism of the unique micro/nano flower structure of AZO film was proposed as below. Normally, because (002) polar plane of ZnO has the highest atomic packing density, and thus *c* axis is the thermodynamically favorable growth direction of the ZnO with wurtzite structure under normal crystallization conditions. Therefore the aligned nanorods array structure was common micro-topography for the ZnO or AZO material (Fig. 4a). The preferred orientation and the guiding effects of the AZO seed layer play a significant role during the process of growth and evolution of the followed AZO film. In this work, the (100) dominant plane of the AZO seed layer is special and critical factor for the subsequent growth. In fact, orientation control of ALD thin film growth is much complex and affected by many factors such as pressure, precursor and temperature etc^28^.

**Figure 5:**
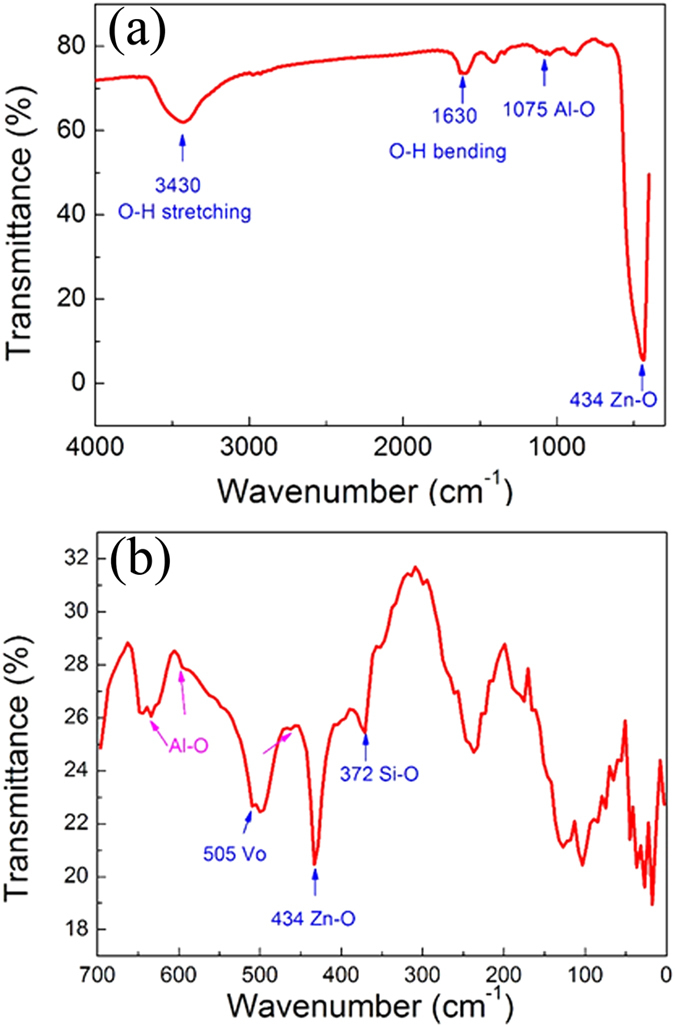
The FT-IR at thin film reflecting measurement mode spectra of the AZO ALD seed layer at middle infrared range (**a**) and at far infrared range (**b**).

In ALD method, methyl and ethyl groups have been involved in AZO film growth process (Fig. 4b). Due to the adsorption of the ethyl groups on (002) crystallographic plane of AZO, the *c*-axis crystal growth of AZO may be suppressed^17^,^29^. Furthermore, the methyl groups aggravate such suppression on (002) plane and thus reinforce the preferential growth of (100) crystallographic plane. As a result, the as-prepared AZO seed layer using the ALD technique exhibits (100) dominant plane, which is in agreement with the XRD patterns of the seed layer. In the process of ALD AZO film growth, methyl and ethyl groups play a role in suppression for the *c*-axis crystal of AZO, but will totally be replaced by H_2_O in every reaction cycles and then are removed by the purge gas. In order to confirm the results, the FT-IR measurements at the middle and far infrared wave bands has been adopted to explore the bonding features in the resulting ALD AZO film. Figure 5a presents the FT-IR spectrum of AZO seed layer at middle infrared range. The bands at 3430 and 1630 cm^−1^ were assigned to the stretching and bending vibration of surface -OH groups. The peak at 434 cm^−1^ and the weak band observed at 1075 cm^−1^ corresponds to the Zn—O and Al-O bond stretching vibration^30^. In the spectrum, the C-H stretching mode of methyl and ethyl group in the region around 2900 cm^−1^ is not observed. In order to obtain some information about AZO seed layer, the FT-IR spectrum of AZO film at the far infrared range is shown in Fig. 5b, where the sharp Zn—O peak also can be obtained at 434 cm^−1^. The significant spectroscopic bands at 643, 595, and 461 cm^−1^ appear, which are identified to be the characteristic absorption bands of Al-O. The bands at 505 and 372 cm^−1^ were assigned to the oxygen deficiency in ZnO and the Si-O bond of the substrate material, respectively^31^.

According to the synergistic reaction mechanism of the precursors, a seed layer with the desired dominant orientation can be obtained controllably, which further guides the self-assemble growth of the AZO top layer using the hydrothermal method. Actually, the nanorods array structure and nano flowers were two kinds of typical morphology for the hydrothermal AZO, which can be obtained under condition of the low and high growth temperature, respectively^32^. In ZnO wurtzite structure, each Zn^2+^ ion is surrounded by four O^2−^ ions and vice versa. The asymmetric distribution of the zinc and oxygen atoms along the c axis determines the positive and negative sides of the crystal. The (002) plane is the positive side, which is the exposed surface of zinc atoms, and (00–2) plane is the negative side with the exposed surface of oxygen atoms. The growth units Zn(OH)_4_^2−^ were negatively charged, which should be easily superimposed on the positive side of ZnO crystals. When the temperature of reaction was low, HMTA was able to slowly release OH^−^ groups, thus making the low-concentration of OH^−^ assist in forming nanorods easily and the OH^−^ hardly affect the morphology. However the higher hydrothermal temperature speeds up the releasing rate of OH^−^ groups from HMTA, and more OH^−^ groups can dissolve the (002) plane of ZnO crystal, then the ZnO nanosheets and flower begin to form^14^.

In the hydrothermal growth, for the lower temperature around 95 °C, the nano-rods array AZO structure had been fabricated and presents hydrophobicity with CA ~ 130° (Figure S6). The (002) dominant planes generally lead to the nanorods array structure along [0001] direction, and resulting film still needs the further fluorination method to build the superhydrophobic surface^33^. In contrast, the AZO nucleates and assembles along (100) planes instead of (002) planes, and thus the unique complex flower-like superhydrophobic structures can be formed around 140 °C.

Additionally, HMTA concentrations in solution are an important factor for the hierarchical structure in the hydrothermal process. In this present work, a 1:1 ratio of zinc nitrate hexahydrate (Zn(NO_3_)_2_·6H_2_O) and hexamethylenetetramine (HMTA) were employed in the reaction nutrient solution to meet the reaction requirement. Four different HMTA concentrations 25 mM/L, 50 mM/L, 75 mM/L and 100 mM/L were used to participate the AZO hydrothermal structure growth (Figure S7). The results indicate that high HMTA concentrations will increase the nucleation rate of the AZO crystal grains. Smaller contact angel ~140° was obtained for lower HMTA concentrations of 25 mM/L. On the other hand, lower optical transmittance ~60% happened in higher concentrations of 75 mM/L and 100 mM/L. For the balance of the superhydrophobic and transparent properties of the films, 50 mM/L were chosen as optimal concentrations of HMTA.

**Figure 6:**
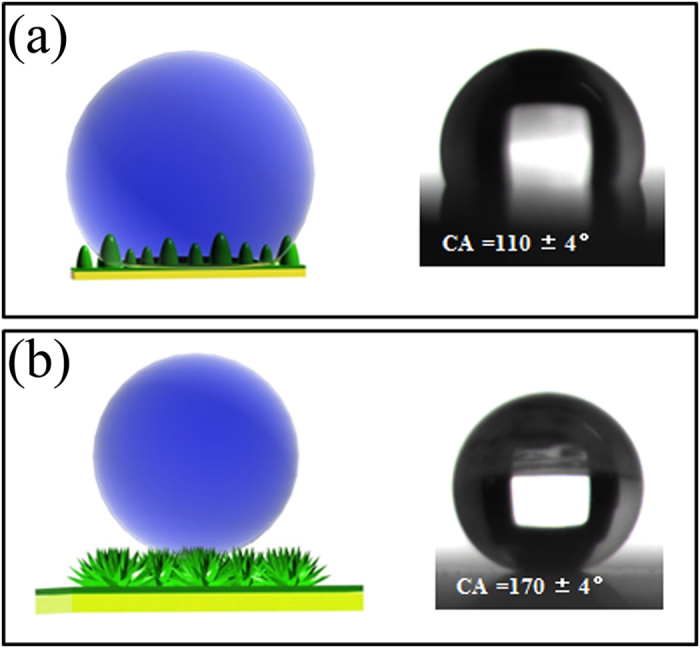
Wetting models and photos of a droplet on rough substrates The AZO ALD film (a) and the AZO hydrothermal film (b).

The schematic pictures of the AZO seed layer and the hydrothermal film with hydrophobic properties are shown in Fig. 6. The CA value of 110 ± 4° was obtained for AZO seed layer, which can be attributed to the lower surface energies of ALD film. Owing to the peony-like morphology with a combination of micro-sized flowers and petals in nano-sized, the AZO hydrothermal film displays the excellent superhydrophobic property with the CA of 170 ± 4° without any other treatment, which is better than other reported values of 158° and 160° using fluoridation method on inorganic materials^5^,^16^.

The different hydrophobic properties between the AZO seed layer and the AZO hydrothermal film can be explained by classical wetting theories^34^. The hydrophobic property of rice-like AZO seed layer was a good example of Wenzel model (Fig. 6a), in which liquids are assumed to completely fill the grooves of rough surfaces, and the apparent contact angle *θ*_*W*_ follows equation: cos*θ*_*W*_ = *r*cos*θ*_*Y*_ (*θ*_*Y*_ is the contact angle, and *r* is the surface roughness factor, defined as the ratio between the actual surface area and the apparent surface area of the rough surface).

The hydrophobicity of the AZO hydrothermal film was greatly increased. The micro- and nano-structures of this AZO film induced transitions between Wenzel and Cassie states (Fig. 6b). The great number of varied air pockets among the flowers and petals support the water droplet and thus further improves the superhydrophobic property of the surface. In this case, Cassie state can be derived as cos*θ*_*C*_ = *f*_*s*_ (cos*θ* + 1) − 1, where *θ*_*C*_ is the apparent contact angle and *f*_*s*_ is the fractional area of the given solid surface with a contact angle of *θ*.

On the other hand, the polar (002) planes are metastable and have high surface energy, while the nonpolar (100) planes are stable and have lower surface energy, which is beneficial to the fabrication of the superhydrophobic surface. According to the experimental results and theories models, the high ratio of (100)/(002) AZO hydrothermal film with the micro-sized flowers and nano-sized petals presents an excellent superhydrophobic property, which could be a model for future design and development of the superhydrophobic surfaces.

The durability of the superhydrophobic film is important for the practical applications. The CA of the AZO hydrothermal film was measured every month after storage under ambient condition for a period of time. The superhydrophobicity of the film remained excellent, as indicated by the constant CA values for 5 months. The durable superhydrophobicity results from the stable chemical composition and structure of the inorganic material. The studies on the superhydrophobicity of the AZO films with different hierarchical structures are ongoing and the results will be reported soon.

## Conclusion

A simple, inexpensive and controllable two-step layer-by-layer process using the ALD and hydrothermal methods was applied to fabricate transparent and durably superhydrophobic (CA = 170 ± 4°) inorganic micro/nano hierarchical films. The bottom homogeneous AZO seed layer controlled the subsequent growth of AZO film along (100) preferred planes with micro/nano scale peony-like structure. This is the first report about fabrication of the transparent and superhydrophobic coatings through the guiding effect of the ALD seed layer instead of using the expensive fluorinated reagents. Due to the controllable crystalline phase and special morphology of the AZO layer obtained by the ALD and hydrothermal methods, the current strategy is suitable for numerous other inorganic oxide materials and substrates with special shapes, and promises a potential foreground in various areas.

## Methods

### Preparation of AZO seed layer by ALD

Substrates were cleaned by acetone, alcohol and distilled water, respectively. An ALD system (TALD-150 Kemicro Jiaxing) was employed to deposit AZO films on substrates. Diethyl zinc [DEZn, Zn(C_2_H_5_)_2_], trimethyl aluminum (TMAl, Al(CH_3_)_3_) and distilled-water (H_2_O) were used as precursors of Zn, Al, and O, respectively. DEZn, TMAl, and H_2_O were fed into the chamber through separate inlet lines and purged by N_2_ gas in ALD cycles. The pulse sequence of ZnO was DEZn - N_2_ purge - H_2_O - N_2_ purge, and the dose time and purge time was 0.02-25-0.015-25 s. The Al doping sequence in ZnO was DEZn - N_2_ purge - TMAl - N_2_ purge -H_2_O - N_2_ purge, and the dose time and purge time was 0.01-25-0.02-25-0.015-25 s. The sequence ratio of Zn:Al was 19:1 and the growth rate of AZO is 1.5 Å per cycle at 150 °C and 0.15 Torr.

### Preparation of AZO micro flower structure composite film via hydrothermal method

The substrates with AZO seed film were placed flat on the bottom of a 50 mL Telfon-lined stainless steel autoclave. 0.5652 g Zn (NO_3_)_2_·6H_2_O, 0.0375 g Al (NO_3_)_3_·9H_2_O (5% atomic concentration doped) and 0.2668 g C_6_H_12_N_4_ (HMTA) were dissolved in 40 mL of deionized water and poured into the autoclave. Hydrothermal synthesis was carried out at 140 °C for 4 h. The autoclave was cooled down to room temperature and the sample was washed repeatedly with ethanol, deionized water and dried with nitrogen.

### Materials characterization

The surface morphology and root mean square (RMS) roughness of the AZO seed films were investigated by an atomic force microscopy (AFM) (Asylum Research MFP-3D-SA). X-ray Photoelectron Spectroscopy (XPS, TSC K-Alpha, AlKa) was adopted to investigate the elemental states of Zn and Al. The concentration of Al was determined by using inductively coupled plasma atomic emission spectroscopy (ICP-AES, Perkin-Elmer, Optima 2100DV. The morphology of the composite films was observed in a scanning electron microscope (E-SEM; Quanta 250, FEG) and transmission electron microscopy (TEM; Tecnai G2). Crystal properties of the AZO seed layer film and films were characterized by grazing-incidence x-ray diffraction (GI-XRD) using Cu-Kα radiation of 0.15406 nm. The fourier transform infrared spectra (FTIR) were recorded using a Thermo Fisher IS 50 spectrophotometer in the wave number range of 0–4000 cm^−1^ at room temperature. WCA measurement was carried out with a CA101 contact angle goniometer using a 4-μL water droplet.

## Additional Information

**How to cite this article**: Li, Y. *et al.* Micro/Nano hierarchical peony-like Al doped ZnO superhydrophobic film: The guiding effect of (100) preferred seed layer. *Sci. Rep.* **6**, 19187; doi: 10.1038/srep19187 (2016).

## Supplementary Material

Supplementary Information

## References

[b1] Yu E. *et al.* Extreme wettability of nanostructured glass fabricated by non-lithographic, anisotropic etching. Sci. Rep 5, 9362 (2015). 25791414 10.1038/srep09362 PMC4366763

[b2] Xu L. G., Geng Z., He J. H. & Zhou G. Mechanically robust, thermally stable, broadband antireflective, and superhydrophobic thin films on glass substrates. ACS Appl. Mater. Interfaces 6, 9029–9035 (2014). 24848810 10.1021/am5016777

[b3] Liu K. S. *et al.* Bio-inspired titanium dioxide materials with special wettability and their applications. Chem. Rev 114, 10044–10094 (2014). 24956456 10.1021/cr4006796

[b4] Jiang L., Zhao Y. & Zhai J. A lotus-leaf-like superhydrophobic surface: A porous microsphere/nano fiber composite film prepared by electrohydrodynamics. Angew. Chem. Int. Ed 43, 4338–4341 (2004). 10.1002/anie.200460333 15368387

[b5] Liu Y. *et al.* Reversible superhydrophobic superhydrophilic transition of ZnO nanorod/epoxy composite films. ACS Appl. Mater. Interfaces 4, 3959–3964 (2012). 22764733 10.1021/am300778d

[b6] Lee M., Kwak G. & Yong K. Wettability control of ZnO nanoparticles for universal applications. ACS Appl. Mater. Interfaces 3, 3350–3356 (2011). 21819107 10.1021/am2004762

[b7] Wang Z. L. & Song J. H. Piezoelectric nanogenerators based on Zinc Oxide nanowire arrays. Science 312, 242–246 (2006). 16614215 10.1126/science.1124005

[b8] Chu S. *et al.* Electrically pumped waveguide lasing from ZnO nanowires. Nature Nanotech 6, 506–510 (2011). 10.1038/nnano.2011.97 21725304

[b9] Ren J. B. *et al.* Novel fabrication of TiO_2_/ZnO nanotube array heterojunction for dye-sensitized solar cells. RSC Adv 4, 7454–7460 (2014).

[b10] Ruoho M. *et al.* Influence of aluminium doping on thermoelectric performance of atomic layer deposited ZnO thin films. Appl. Phys. Lett 103, 203903 (2013).

[b11] Minami T. Transparent conducting oxide semiconductors for transparent electrodes. Semicond. Sci. Technol 20, 35–44 (2005).

[b12] Haghdoost A. & Pitchumani R. Fabricating superhydrophobic surfaces via a two-step electrodeposition technique. Langmuir 30, 4183–4191 (2014). 24083366 10.1021/la403509d

[b13] Li B. J. *et al.* Reversible wettability control of ZnO thin films synthesized by hydrothermal process on different buffer layers. Mater. Lett 110, 160–163 (2013).

[b14] Chen Z. W. *et al.* Sol-gel-hydrothermal synthesis and conductive properties of Al-doped ZnO nanopowders with controllable morphology. J. Alloys. Comp 587, 692–697 (2014).

[b15] Mohanta A. *et al.* Effect of pressure and Al doping on structural and optical properties of ZnO nanowires synthesized by chemical vapor deposition. J. Lumin 146, 470–474 (2014).

[b16] Gao Y. Q. *et al.* Highly transparent and UV-resistant superhydrophobic SiO_2_ coated ZnO nanorod arrays. ACS Appl. Mater. Interfaces 6, 2219–2223 (2014). 24495100 10.1021/am405513k PMC3985694

[b17] Pung S. Y. *et al.* Preferential growth of ZnO thin films by the atomic layer deposition technique. Nanotechnology 19, 435609 (2008). 21832704 10.1088/0957-4484/19/43/435609

[b18] Wisdom K. M. *et al.* Self-cleaning of superhydrophobic surfaces by self-propelled jumping condensate. Proc. Natl. Acad. Sci. USA 110, 7992–7997 (2013). 23630277 10.1073/pnas.1210770110 PMC3657783

[b19] Chen J. T. *et al.* The effect of Al doping on the morphology and optical property of ZnO nanostructures prepared by hydrothermal process. Appl. Surf. Sci. 255, 3959–3964 (2009).

[b20] Chen M. *et al.* X-ray photoelectron spectroscopy and auger electron spectroscopy studies of Al-doped ZnO films. Appl. Surf. Sci. 158, 134–140 (2000).

[b21] Fan J. C. C. & Goodenough J. B. X-ray photoemission spectroscopy studies of Sn-doped indium-oxide films. J. Appl. Phys. 48, 3524 (1977).

[b22] Lee D. J. *et al.* Structural and electrical properties of atomic layer deposited Al-doped ZnO films. Adv. Funct. Mater. 21, 448–455 (2011).

[b23] Qian X. *et al.* Atomic layer deposition of Al-doped ZnO films using aluminum isopropoxide as the Al precursor. Chem. Vapor Depos. 19, 180–185 (2013).

[b24] Sengupta J. *et al.* Influence of annealing temperature on the structural, topographical and optical properties of sol–gel derived ZnO thin films. Mater. Lett. 65, 2572–2574 (2011).

[b25] Nilsen O. *et al.* Simulation of growth dynamics for nearly epitaxial films. J. Cryst. Growth 308, 366–375 (2007).

[b26] Ueno N. *et al.* Low-temperature hydrothermal synthesis of ZnO nanosheet using organic/inorganic composite as seed layer. Mater. Lett 86, 65–68 (2012).

[b27] Sui C., Lu Z. & Xu T. Effects of annealing temperature on photoluminescence of ZnO nanorods hydrothermally grown on a ZnO:Al seed layer. Opt. Mater. 35, 2649–2653 (2013).

[b28] Wang X. R. *et al.* Atomic layer deposition of metal oxides on pristine and functionalized graphene. J. Am. Chem. Soc 130, 8152–8153 (2008). 18529002 10.1021/ja8023059

[b29] Tynell T. & Karppinen M. Atomic layer deposition of ZnO: a review. Semicond. Sci. Technol 29, 043001 (2014).

[b30] Zhao J. *et al.* Synthesis and photoluminescence properties of ZnO powder by solution combustion method. J. Mater. Sci.: Materials in Electronics 22, 1361–1365 (2011).

[b31] Djelloul A. *et al.* Photoluminescence, FTIR and X-ray diffraction studies on undoped and Al-doped ZnO thin films grown on polycrystalline α-alumina substrates by ultrasonic spray pyrolysis. J. Lumin. 130, 2113–2117 (2010).

[b32] Zhang X. L. *et al.* Surface-morphology evolution of ZnO nanostructures grown by hydrothermal method. Crystal Research and Technology 49, 220–226 (2014).

[b33] Malm Jari. *et al.* Photo-controlled wettability switching by conformal coating of nanoscale topographies with ultrathin oxide films. Chem. Mater. 22, 3349–3352 (2010).

[b34] Wenzel R. N. Resistance of solid surfaces to wetting by water. Ind. Eng. Chem. Res 28, 988–994 (1936).

