# Supplementary material for: Micro/Nano hierarchical peony-like Al doped ZnO superhydrophobic film: The guiding effect of (100) preferred seed layer
Source: Sci Rep. 2016 Jan 12;6:19187. doi: 10.1038/srep19187 (PMC4709682; doi:10.1038/srep19187)
Supplement: Supplementary Information [file srep19187-s1.doc]

Micro/Nano hierarchical peony-like Al doped ZnO superhydrophobic film: The guiding effect of (100) preferred seed layer

Yang Li^1,＋^, Jingfeng Wang^1,＋^, Yi Kong^1^, Jia Zhou^1^, Jinzhu Wu^1^, Gang Wang^1^, Hai Bi^1^, Xiaohong Wu^1^,* , Wei Qin^2^,* and Qingkun Li^3^

^1^Department of Chemistry, Harbin Institute of Technology, Harbin, Heilongjiang 150001, PR China

^2^School of Materials Science and Engineering, Harbin Institute of Technology, Harbin, Heilongjiang 150001, PR China

^3^Key Laboratory of Electrical Engineering, College of Heilongjiang Province Major Laboratories of Integrated Circuits, Heilongjiang University

*Correspondence and requests for materials should be addressed to X.H.W. (email: wuxiaohong@hit.edu.cn) or W.Q. (email: qinwei@hit.edu.cn)

^+^These authors contributed equally to this work

**Supporting Information**

**1. The detailed schematic of fabrication the hierarchically structured superhydrophobic AZO thin films.**


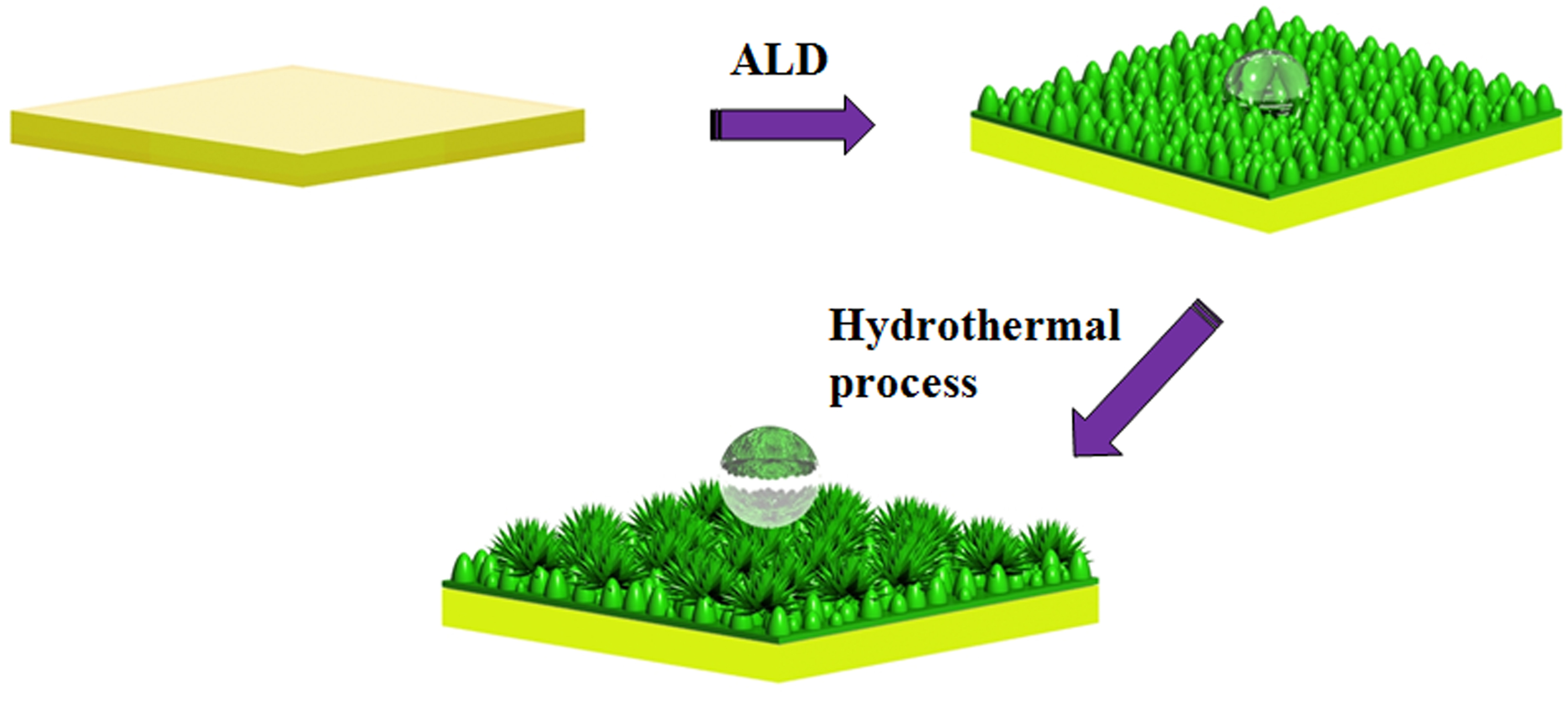


**Figure S1.** Schematic drawing of the fabrication of superhydrophobic micro-nanostructured Al doped ZnO thin films using ALD method and the hydrothermal technique. Nano-rice (CA of 110 ± 4°) and micro-peony (CA of 170 ± 4°) like surface features of superhydrophobic Al doped ZnO films are formed through a two-step process combining the atomic layer deposition (ALD) and the hydrothermal technique.

**2. The distribution of Al dopants in the surface was also investigated using energy-dispersive x-ray spectroscopy (EDS). It can be found that the atomic concentration of Al is 3.41 % for the AZO seed layer.**


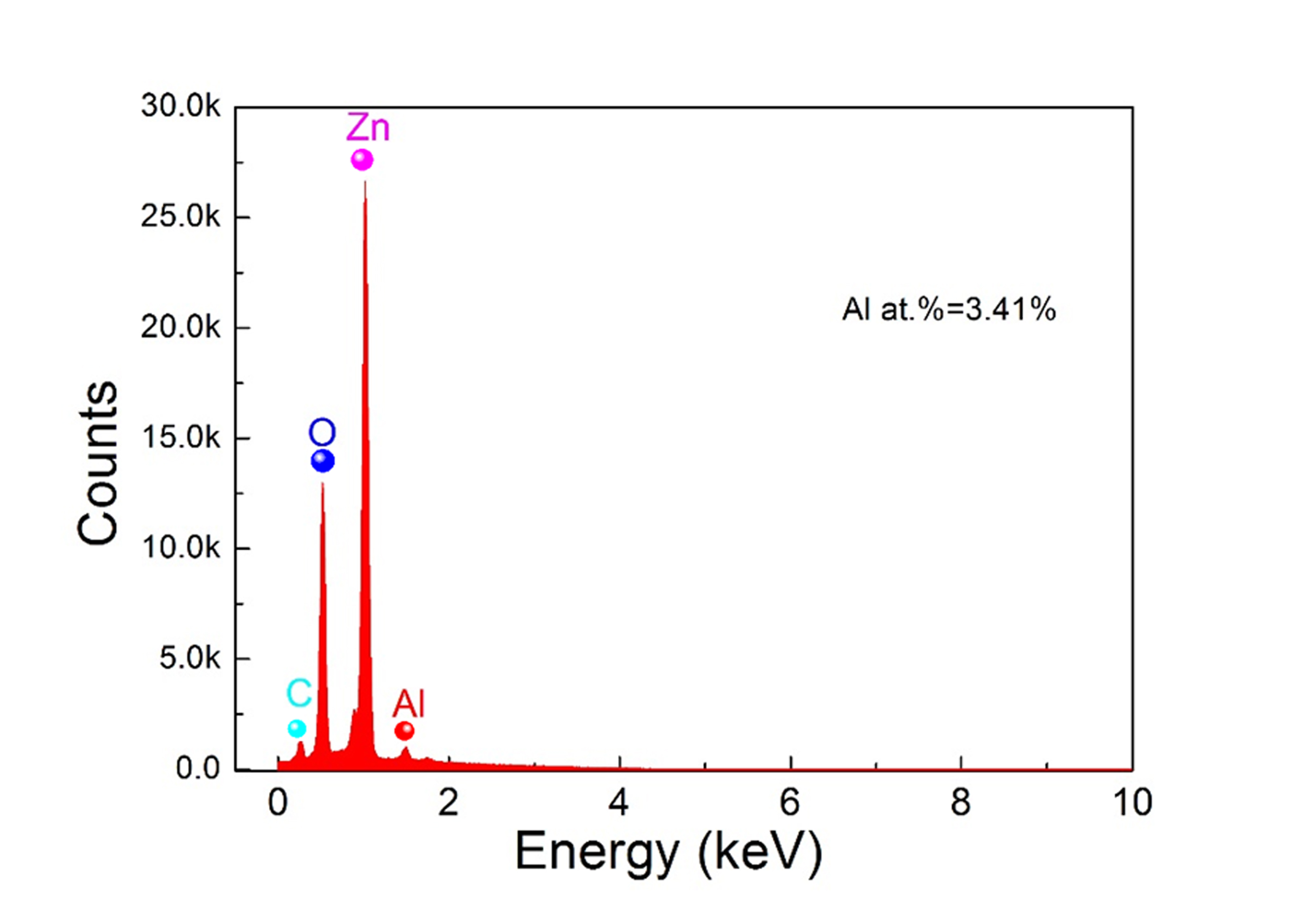


**Figure S2.** SEM-EDS result of the ALD AZO seed layer.

**3. The transmittance spectra of the AZO seed layer and the hydrothermal layer indicate that the average transmittance is about 95% and 80% for the AZO 20 nm seed layer and the AZO hydrothermal layer, respectively.**


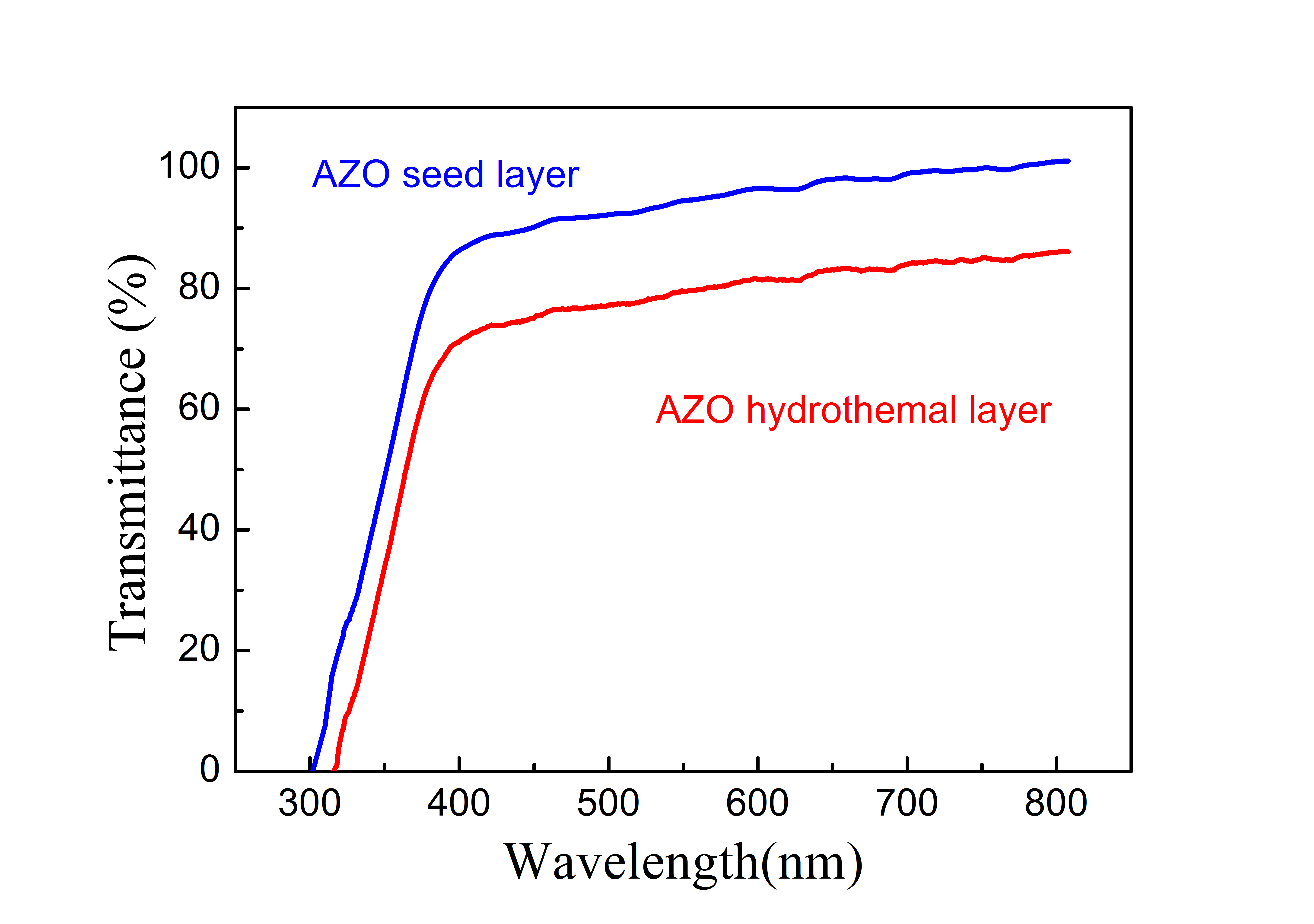


**Figure S3.** Optical transmittance of AZO seed layer and hydrothermal layer.

**4. The optical band gap (E*_g_*) of the AZO seed layer and the hydrothermal layer were estimated by Tauc relation:**


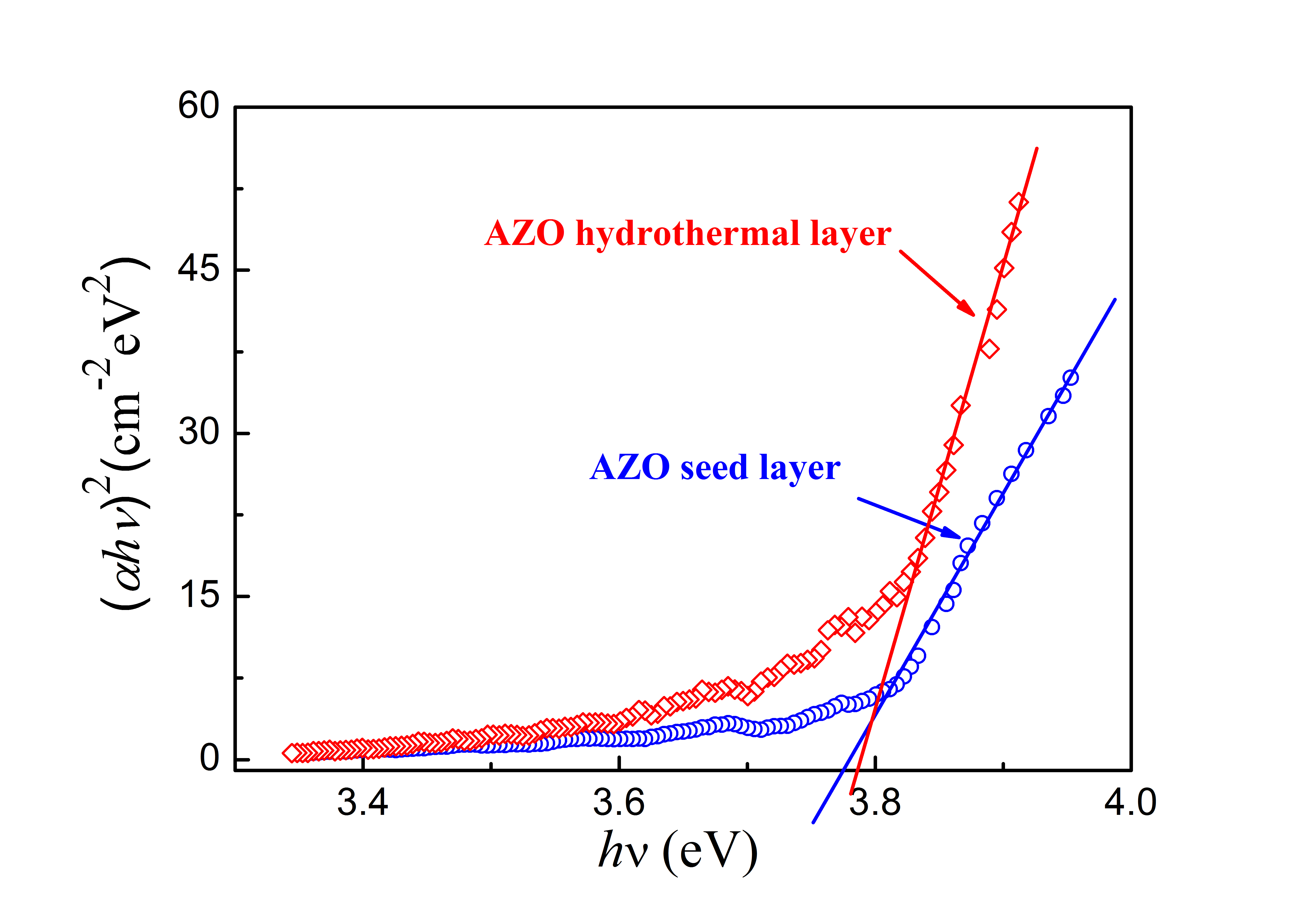


**Figure S4.** Plots of (*αhν*)^2^ against (*hν*) for AZO seed layer and hydrothermal layer.

**5. The chemical states of the compositional elements in AZO hydrothermal film were revealed by the XPS.**

**
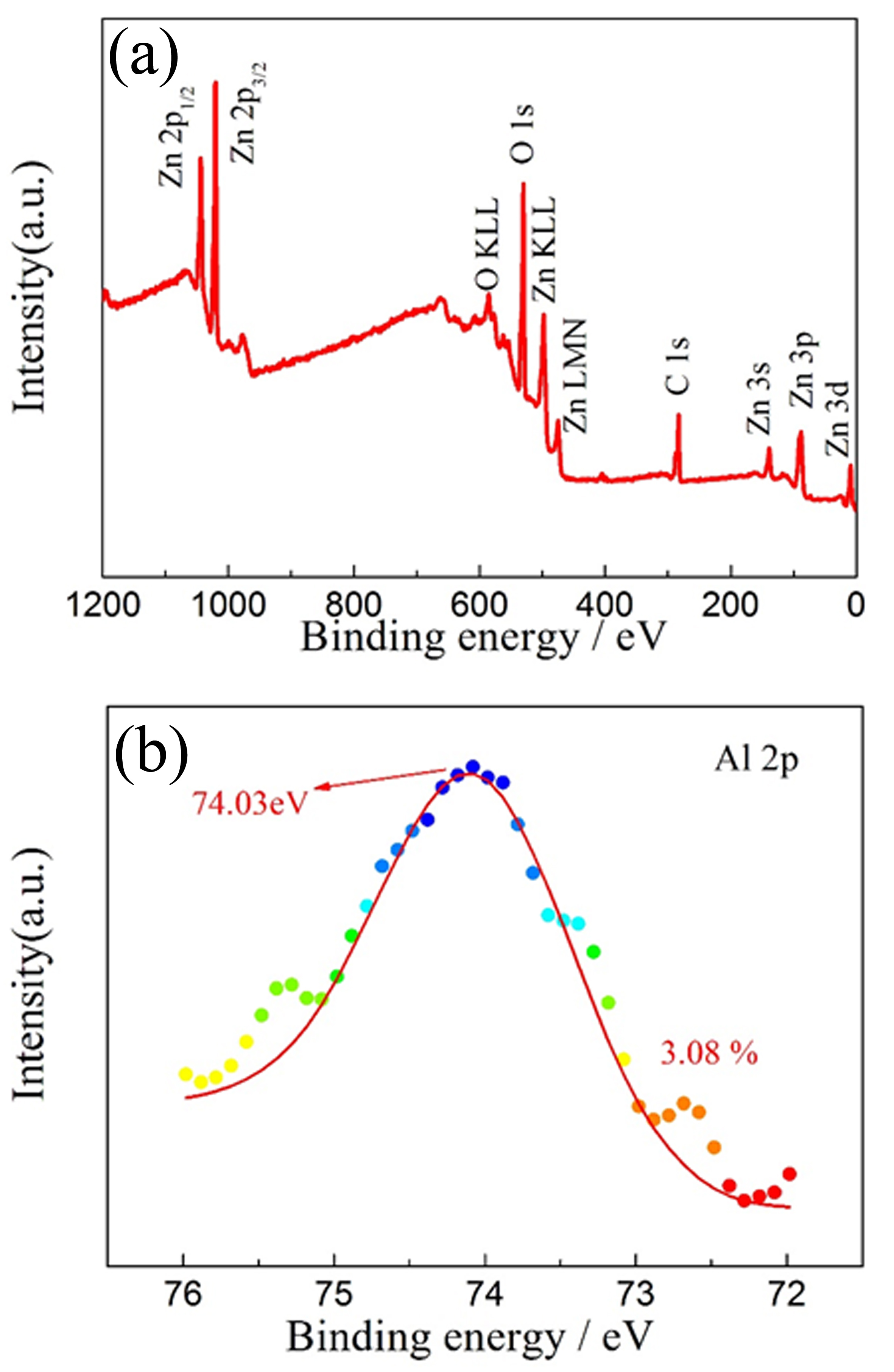
**

**Figure S5.**  (a) Survey XPS spectrum of the AZO hydrothermal film. (b) High resolution XPS spectrums of the Al 2P core level regions and their Gaussian-resolved results.

**6. In the hydrothermal growth, for the temperature around 95℃, the nano-rods array AZO structure had been fabricated and presents hydrophobicity with CA~130°**


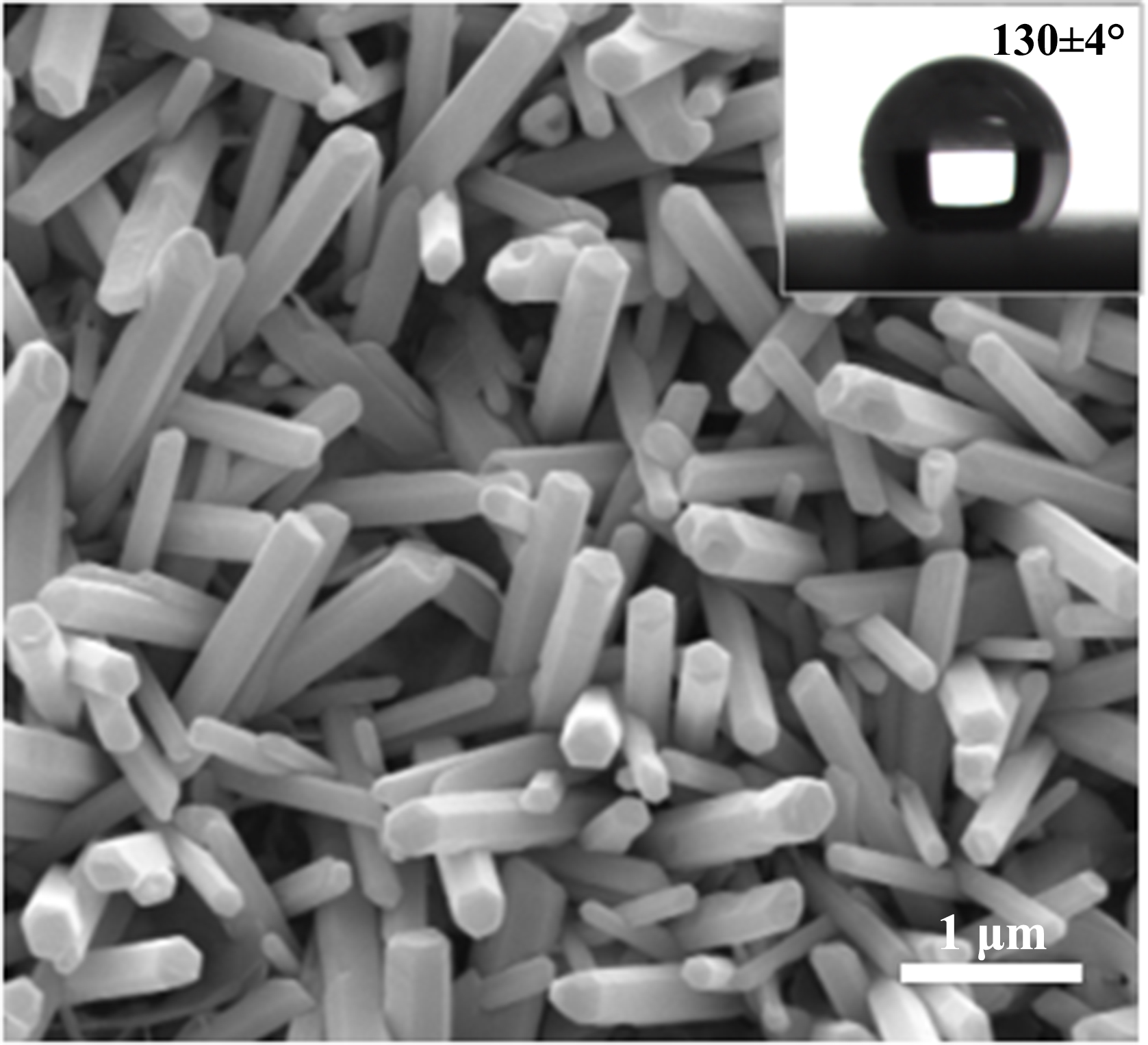


**Figure S6.** SEM top-images of the nano-rods array AZO structure. The insert is the Wetting models and photos of a droplet on the film.

**7. Four different HMTA concentrations 25 mM/L, 50 mM/L, 75 mM/L and 100 mM/L had been employed to participate the AZO hydrothermal growth.**


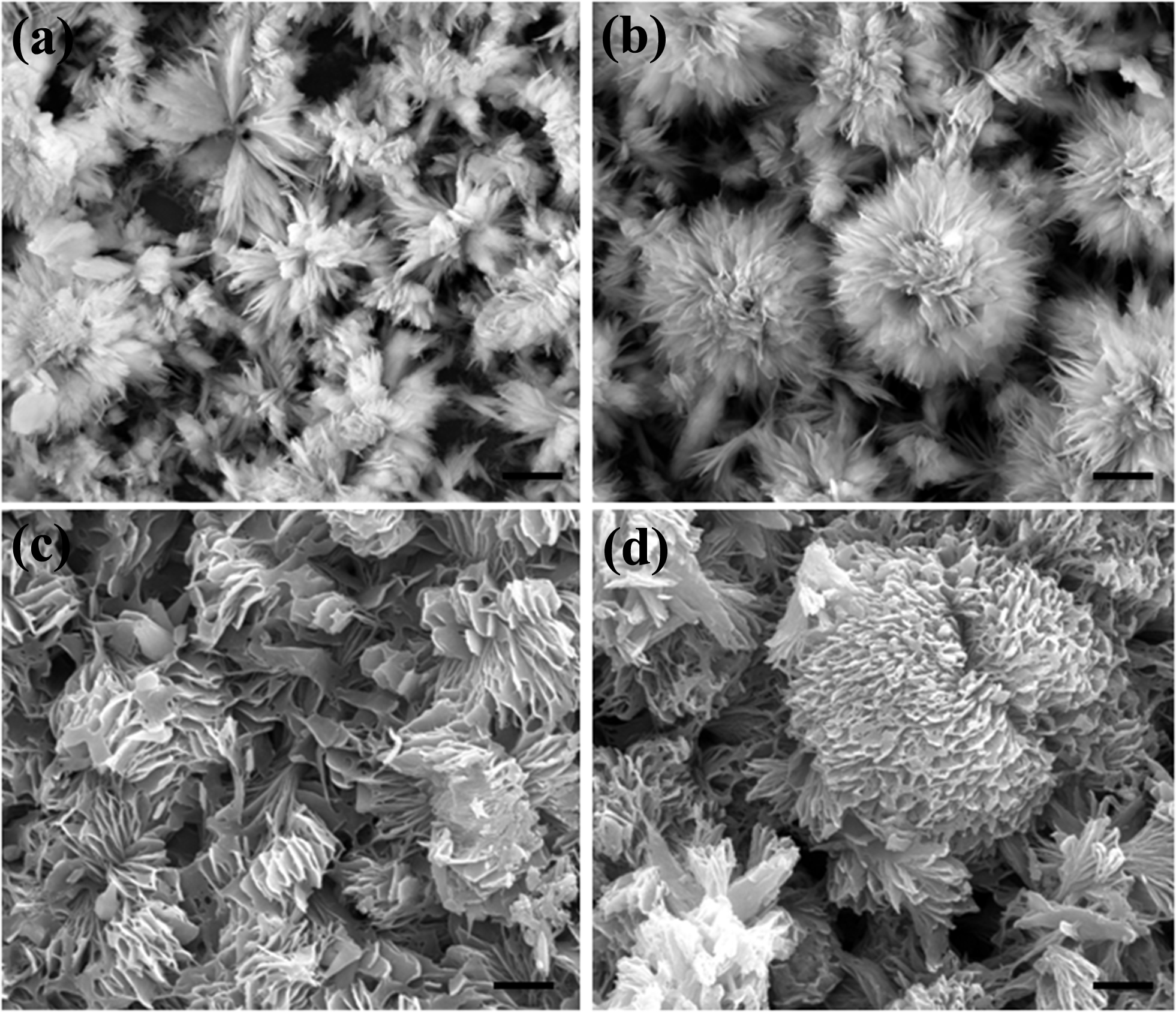


**Figure S7.** SEM top-images of the AZO hydrothermal flower like film growth under different HMTA concentrations (a) 25 mM/L, (b) 50 mM/L, (c) 75 mM/L and (d) 100 mM/L, respectively; The scale bar is equal to 1μm.
